# Care planning needs of palliative home care clients: Development of the interRAI palliative care assessment clinical assessment protocols (CAPs)

**DOI:** 10.1186/1472-684X-13-58

**Published:** 2014-12-15

**Authors:** Shannon Freeman, John P Hirdes, Paul Stolee, John Garcia, Trevor Frise Smith, Knight Steel, John N Morris

**Affiliations:** School of Health Sciences, University of Northern British Columbia, 3333 University Way, Prince George, British Columbia V2N 4Z9 Canada; School of Public Health and Health Systems, University of Waterloo, 200 University Ave. West, Waterloo, ON N2L 6P4 Canada; Department of Sociology, Nipissing University, North Bay, Ontario Canada; Retired Chief Emeritus of Geriatrics, Hackensack University Medical Center, 20 Prospect Ave, Hackensack, NJ 07601 USA; Hebrew Senior Life, 1200 Centre Street, Boston, MA 02131 USA

## Abstract

**Background:**

The interRAI Palliative Care (interRAI PC) assessment instrument provides a standardized, comprehensive means to identify person-specific need and supports clinicians to address important factors such as aspects of function, health, and social support. The interRAI Clinical Assessment Protocols (CAPs) inform clinicians of priority issues requiring further investigation where specific intervention may be warranted and equip clinicians with evidence to better inform development of a person-specific plan of care. This is the first study to describe the interRAI PC CAP development process and provide an overview of distributional properties of the eight interRAI PC CAPs among community dwelling adults receiving palliative home care services.

**Methods:**

Secondary data analysis used interRAI PC assessments (N = 6,769) collected as part of regular clinical practice at baseline (N = 6,769) and follow-up (N = 1,000). Clients across six regional jurisdictions in Ontario, Canada, assessed to receive palliative homecare services between 2006 and 2011 were included (mean age 70.0 years; ±13.4 years). Descriptive analyses focused on the eight interRAI PC CAPs: Fatigue, Sleep Disturbance, Nutrition, Pressure Ulcers, Pain, Dyspnea, Mood Disturbance and Delirium.

**Results:**

The majority of clients triggered at least one CAP while two thirds triggered two or more. Triggering rates ranged from 74% for the Fatigue CAP to less than 15% for the Delirium and Pressure Ulcers CAPs. The hierarchical CAP triggering structure suggested Fatigue and Dyspnea CAPs were persistent issues prevalent among the majority of clients while Delirium and Pressure Ulcers CAPs rarely trigger in isolation and most often trigger later in the illness trajectory.

**Conclusion:**

When any of the eight interRAI PC CAPs are triggered, clinicians should take notice. CAPs triggered at high rates such as fatigue, dyspnea, and pain warrant increased attention for the majority of clients. Consideration of triggered CAPs provide evidence to inform a collaborative decision making process on whether or not issues raised by the CAPs should be addressed in the plan of care. Integrating evidence from the interRAI PC CAPs into the clinical decision making process support care planning to address client strengths, preferences and needs with greater acuity.

## Background

Palliative care prioritizes the management of severe, unpleasant symptoms, especially pain, to improve quality of life (QOL). Quite often, but not always, this effort is directed to the care of persons with a life limiting illness. Palliative care also strives to improve the sense of well-being of the person’s informal support network, including family members and other caregivers [1]. At the most basic level, palliative care may be “achieved through prevention and relief of suffering by means of early identification, comprehensive assessment, and treatment of pain and physical, psychosocial, or spiritual problems” [2].

With support from informal care networks, community based palliative services have made dying at home increasingly accessible to persons with a life limiting illness. Palliative care services provided to meet person-specific needs at the appropriate time in the preferred setting are essential supports for persons with a life limiting illness who prefer a home death. Community based palliative home care has been found to have a positive impact on quality of life and reduce health care expenditures for persons faced with a life limiting illness and their informal support network [3, 4]. Palliative home care programs have also been shown to improve quality of life, reduce physical symptoms, reduce psychological distress, and improve accessibility to formal care providers [5]. Palliative care promotes person-specific care where resources and supports are tailored to meet need on a case-by-case basis.

Standardized assessment tools are crucial to identifying the specific needs of the person. Further, by collecting a substantial number of assessments an evidence base can be developed that can be used to design appropriate approaches to care. To this end, the interRAI Palliative Care (interRAI PC) assessment instrument was developed as part of an integrated suite of instruments spanning the continuum of care [6, 7]. The interRAI assessment instruments use common measurements and common assessment approaches to enable linkage of individual level data as persons transition across the continuum of care (e.g., from home care and long-term care to acute and mental health services) [8–10].

The interRAI PC is a standardized comprehensive assessment tool providing person-specific information to inform the care planning process. Information gathered from the interRAI PC enables assessment of outcomes, tracking of change in person needs over time, quality assessment, and may inform future development of a case mix system for persons receiving palliative care [6]. In Canada, the interRAI suite of assessment instruments are used in multiple care settings including: home care, assisted living, complex continuing care (CCC), LTC, acute care, inpatient and community mental health, and post-acute rehabilitation [11, 12]. The interRAI PC has been newly implemented in Ontario, Canada, joining other mandated interRAI instruments including the RAI-Home Care, RAI-Mental Health, interRAI Community Health Assessment, interRAI Contact Assessment and RAI 2.0 [11–13].

The interRAI PC is used by front line palliative care providers as they seek to address multiple aspects of clinical complications, physical and cognitive decline, as well as social support systems and end of life preferences. The communication of this information between the person and provider of care is geared to ensuring that the unique needs of the person are addressed in an appropriate and timely manner.

The interRAI PC Clinical Assessment Protocols (CAPs) focus on specific clinical, functional, and life quality issues [7]. Using algorithms embedded within the interRAI PC, the CAPs alert the assessor to specific problems and indicate either risk of their appearance or potential for improvement, if present. Both can be addressed in the care plan [14]. Each CAP contains four components: issue statement, goals of care, triggers, and guidelines. The issue statement provides a clear rationale for why the specific CAP domain should be an important part of the palliative care services under consideration and examines the impact of the clinical issue on the person’s life. The goals of care highlight the benefits of potential intervention [15–18]. These vary by CAP and may include: reducing distress, resolving the problem in its entirety, reducing the risk of deterioration, eliminating side effects of an intervention, or increasing the opportunity to improve or maintain function when possible. Targeting triggers (also embedded within the instrument) have been created based on a large palliative care dataset to identify which persons appear to be most likely to benefit from an intervention.

Detailed technical information on the statistical code for the CAP triggers may be accessed via http://www.interRAI.org. Best practice care guidelines summarize what are likely the most appropriate responses to the issue. By outlining various approaches to the problem, clinicians are able to consider underlying issues and treatment alternatives when creating a person-specific plan of care. The CAP manual includes additional resources and reference materials enabling quick access to more detailed information.

The first set of eight interRAI PC CAPs released in 2013 address the following domains: Fatigue, Sleep Disturbance, Nutrition, Pressure Ulcers, Pain, Dyspnea, Mood Disturbance, and Delirium [7]. These CAPs are distinct from other CAPs, such as those accompanying the interRAI home care, long-term care, and mental health instruments. These CAPs allow for prioritizing the person’s needs as death approaches. The CAPs highlight areas of need that may benefit from treatment or targeted care, even in the final stages of life. Previously, benefits of the CAPs for both risk assessment and care planning in the community and in institutional mental health settings have been documented [19, 20]. Moreover, the benefits for clinicians to use the CAPs to assist in identifying at-risk residents residing in long-term care facilities has also been highlighted [21].

This paper provides the first description of the strengths and limitations of the interRAI PC Clinical Assessment Protocols (CAPs). An overview of the CAP development process will be provided, as well as an examination of how the CAPs function in relation to physical, psychological, and social characteristics.

## Methods

### CAP development process

CAP development entailed a three phase multi-year process conducted by an international committee with members from nine countries. The committee considered evidence from peer-reviewed literature and international best practice guidelines [7]. Phase one focused on a review of best practice guidelines for each CAP domain area, gathered from at least three global regions. The focus of the CAP domain was then identified. When guidelines were unavailable, relevant peer-reviewed publications were reviewed. During phase two, consultation with subject-matter experts from around the world was undertaken. Direct evaluation of the CAPs by palliative care providers was conducted to support face validity. Responses from both interRAI and outside experts supported the premise that each CAP captured accurate and clinically relevant information. Recommendations during this consultation process were incorporated into the CAP frameworks. Phase three focused on creation of triggering algorithms based on an analysis of Canadian data. The interRAI PC CAP manual was developed, detailing information on trigger rates, factors associated with triggering, and best practice guidelines [7].

### Data source

The interRAI PC assessment instrument includes more than 280 items, covering 75 key areas, grouped into 17 specific domains including demographic and intake information, medical diagnoses and conditions, physical and cognitive functioning, and psycho-social and emotional wellbeing [6, 7]. The instrument helps clinicians to provide person-level care rather than site-specific care and therefore may be employed in multiple care settings such as community-based, hospice, or residential care facilities. Assessments, completed by trained individuals with professional backgrounds, including nursing and social work, consolidate information from direct observation, medical records, and communication with the person, their health team, and their informal support network. Information gathered from the interRAI PC may assist the person and members of their care team, in partnership, to identify, address, and evaluate person-specific care needs. The breadth of information collected provides a comprehensive description of the person. Items contained in the interRAI PC have shown excellent inter-rater and test-retest reliability [6, 19]. Data gathered from interRAI PC assessments provide an evidence base, which when combined with clinical judgment, is useful to inform the development and implementation of care plans tailored to the unique needs of each person.

### Clinical assessment protocols (CAPs)

The Dyspnea CAP and Delirium CAP both have binary triggers (do not trigger/trigger) in comparison to the other six CAPs that have two triggering levels (do not trigger/trigger level one/trigger level two). The Dyspnea CAP identifies persons experiencing shortness of breath and highlights strategies to recognize the onset and severity of symptoms [22]. Persons who are currently experiencing delirium trigger the Delirium CAP that highlights clinical strategies not only to identify and treat symptoms but also to prevent foreseeable complications and to improve QOL [23]. The Fatigue CAP is the most frequently triggered CAP. It differentiates the risk for persons currently or at risk of experiencing fatigue (medium risk-trigger level 1, high risk-trigger level 2) and outlines key considerations to address both causes and symptom reduction [24]. Based on the interRAI Pain Scale, the Pain CAP prioritizes persons experiencing pain (medium-trigger level 1, high-trigger level 2) and provides best practice guidelines for assessment and management strategies [25]. The Mood Disturbance CAP differentiates levels of risk of depression by symptom frequency (single-trigger level 1, multiple-trigger level 2) with a goal to improve psychological well-being [26]. It outlines best practice approaches that address the symptoms and investigate the type of disorder. It then lists key considerations for potential treatment and monitoring of the disorder. The Sleep Disturbance CAP differentiates the potential to improve (moderate-trigger level 1 or high-trigger level 2) among persons experiencing a sleep disturbance. Based upon the presence of a list of reversible issues, the Sleep Disturbance CAP focuses on strategies to reduce the disturbance, increase comfort, and improve functioning [27]. The Nutrition CAP identifies persons who may benefit from education and interventions to optimize energy and protein intake, reduce anxiety about not eating, or who could benefit from interventions addressing hunger [28]. Trigger levels focus on persons with a low body mass index (BMI) and differentiates levels based on absence (trigger level 1) or presence (trigger level 2) of weight loss. The Pressure Ulcers CAP emphasizes the importance of appropriate treatment and identifies potential for improvement (moderate-trigger level 1, high-trigger level 2) for persons with pressure ulcers [29].

### Study sample

De-identified cross-sectional pilot data from 6,769 interRAI PC assessments gathered between 2006 and 2011 from palliative home care clients in Ontario, Canada were included for analysis. When follow-up assessments were available, only the first assessment was included. Data were collected from six Community Care Access Centre’s (CCAC), which serve as the first point of access to community based care across the province. Each CCAC is tasked to coordinate specialized supports, including palliative care, for persons under its jurisdiction and to connect persons requiring care with available services/resources in the person’s home or within the respected community. The six CCAC pilot sites were located across various geographical regions of Ontario from north to south, east to west and ranged from primarily metropolitan urban to more rural and geographically dispersed districts. Sample characteristics are shown in Table 1. Age ranged from 18 to 107 years with a mean age of 70.0 years (SD ±13.4 years), of whom more than 80% reported a diagnosis of cancer (n = 5,875). The majority of persons were rated by clinicians to have an estimated prognosis of greater than 6 weeks, with more 40% (n = 2,310) having an estimated prognosis of greater than six months at the time of the assessment. Only 2% (n = 110) had a prognosis of death being imminent.

**Table 1:** **Sample characteristics of clients receiving palliative home care services 2006–2011, Ontario, Canada (N = 6,769)**

	Total population % (N)
**Age groups**	
18-44	4.3 (288)
45-64	29.9 (2,025)
65-74	25.4 (1,718)
75-84	28.7 (1,943)
85 +	11.7 (795)
**Gender**	
Male	49.1 (3,303)
Female	50.9 (3,418)
**Estimated prognosis**	
Greater than 6 months	41.5 (2,310)
6 weeks to 6 months	48.1 (2,677)
Less than 6 weeks	8.4 (468)
Death Imminent	2.0 (110)
**CCAC Site location**	
Site 1	4.0 (270)
Site 2	47.7 (4,581)
Site 3	14.6 (991)
Site 4	7.5 (510)
Site 5	2.1 (142)
Site 6	4.1 (275)
**Diagnosis**	
Have cancer diagnosis	86.8 (5,875)
*Metastatic*	40.0 (2,710)
*Not Metastatic*	46.8 (3,165)
Do not have cancer	9.8 (666)
Diagnosis unspecified	3.4 (228)

### Analysis

Univariate distributional properties were examined for all eight interRAI PC CAPs and cross tabulations were used to examine the hierarchical triggering structure of the CAPs. Associated covariates including age, gender, estimated prognosis, geographic location, and disease diagnosis, were examined using chi-square to determine significant relationships. The hierarchical analysis also employed chi-square analysis to examine covariates among CAPs. All analyses were performed using SAS Version 9.2 with an alpha level of p < 0.05 for all statistical tests; however, the sample is sufficiently large that in some cases differences may be statistically significant at the level, but of modest clinical importance.

Informed consent was not required for the interRAI PC assessment process because the assessment was used as part of the standard of care in routine clinical practice. The interRAI PC data were deidentified prior to submission to the University of Waterloo in order to ensure that they did not constitute personally identifiable health records. Ethics clearance for the analyses of these secondary data was obtained through the University of Waterloo’s Office of Research Ethics (#19424).

## Results

**Figure 1:**
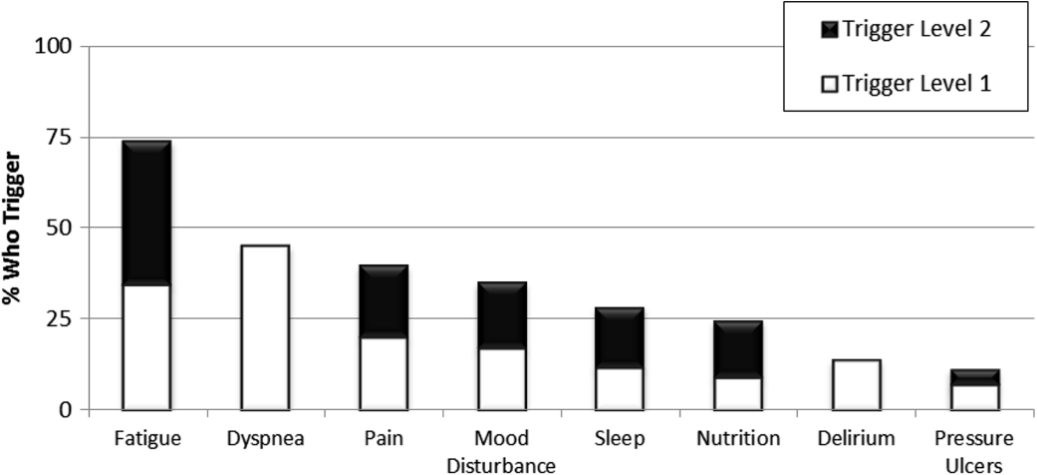
**Triggering rates by CAP of clients receiving palliative home care services 2006–2011, Ontario, Canada (N = 6,769).**

**Figure 2:**
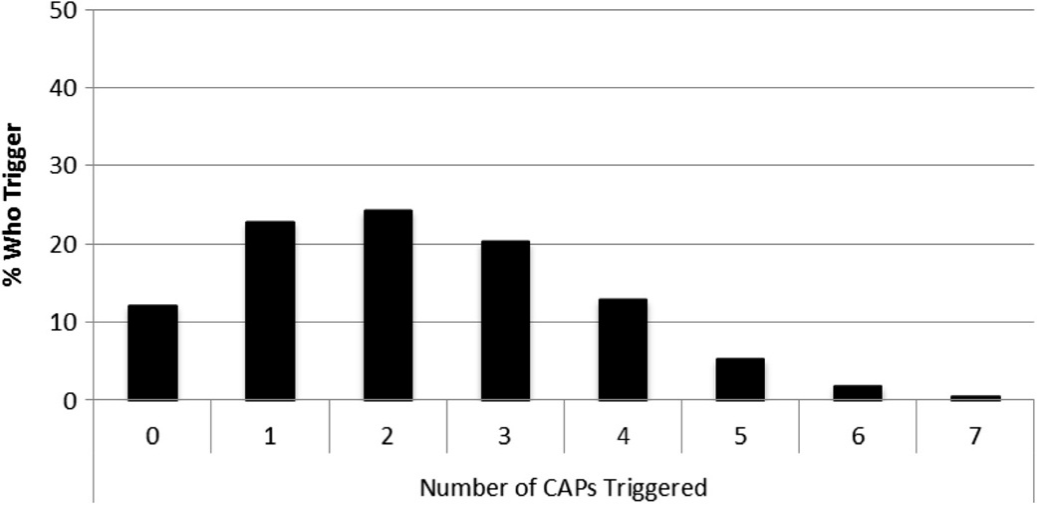
**Number of CAPs triggered by clients receiving palliative home care services 2006–2011, Ontario, Canada (N = 6,769).**

Each CAP contains individualized triggers occurring at different rates from 74% (Fatigue CAP) to less than 15% (Delirium and Pressure Ulcers CAPs) (Figure 1). Spearman’s rank correlations suggested that the majority of CAPs were reasonably independent from each other. Modest correlations were evident between the Fatigue and Delirium CAPs (0.20) and Fatigue and Mood Disturbance CAPs (0.26). Nearly 9 in 10 persons triggered at least one CAP (87.9%, n = 5,950) and approximately two thirds triggered more than two CAPs (Figure 2). Variable distribution differed across the four CAPs dealing with clinical complexity: Dyspnea, Nutrition, Pain, and Pressure Ulcers (Tables 2 and 3), the CAPs dealing with performance: Fatigue and Sleep Disturbance (Table 4), and the cognition/mental health CAPs: Delirium and Mood Disturbance (Table 5). Triggering rates differed by CAP and by geographic location.

**Table 2:** **Distribution of background characteristics by dyspnea and nutrition clinical complexity CAPs of clients receiving palliative home care services 2006–2011, Ontario, Canada (N = 6,769)**

	Dyspnea				Nutrition				
	Not triggered % (N)	Trigger level 1% (N)	Chi-square (df)	p Value	Not triggered % (N)	Trigger level 1% (N)	Trigger level 2% (N)	Chi-square (df)	p Value
**Age groups**									
18-44	68.6 (192)	31.4 (88)	31.8 (4)	<.0001	76.4 (155)	12.3 (25)	11.3 (23)	28.4 (8)	0.0004
45-64	57.1 (1,140)	42.9 (858)			78.7 (1,044)	6.3 (84)	14.9 (198)		
65-74	54.4 (919)	45.6 (769)			76.5 (855)	8.4 (94)	15.1 (169)		
75-84	52.0 (994)	48.0 (919)			73.1 (882)	10.3 (124)	16.7 (201)		
85 +	54.0 (420)	45.9 (356)			71.9 (330)	12.2 (56)	15.9 (73)		
**Gender**									
Male	53.1 (1,727)	46.9 (1,526)	9.6 (1)	0.002	78.9 (1,645)	6.3 (132)	14.8 (308)	38.1 (2)	<.0001
Female	56.9 (1,908)	43.1 (1,446)			72.5 (1,591)	11.4 (251)	16.1 (353)		
**Estimated prognosis**									
Death imminent	37.4 (40)	62.6 (67)	105.2 (3)	<.0001	64.7 (33)	2.0 (1)	33.3 (17)	78.4 (6)	<.0001
Less than 6 weeks	43.8 (203)	56.3 (261)			65.2 (193)	6.8 (20)	28.0 (83)		
6 weeks to 6 months	53.4 (1,413)	46.6 (1,235)			73.5 (1,407)	9.2 (176)	17.3 (331)		
Greater than 6 months	63.7 (1,454)	36.3 (829)			79.4 (1,355)	9.4 (161)	11.2 (191)		
**Geographic location**									
Site 1	62.6 (167)	37.5 (100)	41.5 (5)	<.0001	74.1 (172)	4.7 (11)	21.1 (49)	38.9 (10)	<.0001
Site 2	53.3 (2,389)	46.7 (2,091)			77.1 (1,945)	8.2 (208)	14.7 (371)		
Site 3	58.1 (574)	41.9 (414)			71.3 (627)	10.8 (95)	17.9 (157)		
Site 4	54.0 (273)	46.1 (233)			75.6 (272)	10.8 (39)	13.6 (49)		
Site 5	76.4 (107)	23.6 (33)			72.8 (75)	17.5 (18)	9.7 (10)		
Site 6	56.6 (155)	43.4 (119)			81.4 (175)	5.6 (12)	13.0 (28)		
**Diagnosis**									
Have cancer diagnosis	57.8 (2,295)	42.2 (1,678)	98.9 (3)	<.0001	76.1 (1,912)	8.71 (219)	15.2 (383)	39.5 (6)	<.0001
Have cancer and non-cancer diagnosis	56.5 (1,023)	43.5 (789)			77.8 (1,047)	6.76 (91)	15.5 (208)		
Have non-cancer diagnosis only	37.8 (245)	62.3 (404)			67.5 (241)	16.8 (60)	15.7 (56)		
Diagnosis unspecified	46.2 (103)	53.9 (119)			68.8 (66)	13.5 (13)	17.7 (17)		

Note: df denotes degrees of freedom.

**Table 3:** **Distribution of background characteristics by pain and pressure ulcers clinical complexity CAPs of clients receiving palliative home care services 2006–2011, Ontario, Canada (N = 6,769)**

	Pain					Pressure ulcers				
	Not triggered % (N)	Trigger level 1% (N)	Trigger level 2% (N)	Chi-square (df)	p Value	Not triggered % (N)	Trigger level 1% (N)	Trigger level 2% (N)	Chi-square (df)	p Value
**Age groups**										
18-44	47.4 (120)	20.6 (52)	32.0 (81)	156.7 (8)	<.0001	91.2 (239)	5.34 (14)	3.4 (9)	87.8 (8)	<.0001
45-64	51.9 (955)	23.1 (425)	25.0 (460)			91.6 (1,708)	3.9 (73)	4.5 (84)		
65-74	59.9 (948)	21.2 (336)	18.8 (298)			89.4 (1,414)	5.6 (88)	5.1 (80)		
75-84	67.4 (1,191)	16.2 (286)	16.3 (288)			88.3 (1,573)	8.0 (143)	3.7 (66)		
85 +	70.8 (499)	16.1 (113)	13.1 (92)			83.3 (600)	13.3 (96)	3.3 (24)		
**Gender**										
Male	59.5 (1,775)	19.9 (593)	20.6 (614)	2.8 (2)	0.25	88.6 (2,684)	6.7 (204)	4.7 (141)	2.7 (2)	0.26
Female	61.4 (1,913)	19.5 (608)	19.1 (594)			89.6 (2,811)	6.6 (208)	3.8 (120)		
**Estimated prognosis**										
Death imminent	51.5 (51)	24.2 (24)	24.2 (24)	53.2 (6)	<.0001	76.0 (73)	22.9 (22)	1.0 (1)	139.4 (6)	<.0001
Less than 6 weeks	50.5 (218)	23.6 (102)	25.9 (112)			80.0 (348)	15.9 (69)	4.1 (18)		
6 weeks to 6 months	57.3 (1,454)	22.1 (562)	20.6 (522)			87.9 (2,222)	7.5 (190)	4.6 (115)		
Greater than 6 months	65.2 (1,424)	18.2 (398)	16.6 (363)			92.7 (2,022)	3.5 (76)	3.8 (83)		
**Geographic location**										
Site 1	50.2 (133)	23.8 (63)	26.1 (69)	195.2 (10)	<.0001	87.6 (232)	7.9 (21)	4.5 (12)	34.7 (10)	0.0001
Site 2	59.3 (2,382)	20.9 (838)	19.8 (795)			89.5 (3,653)	6.1 (248)	4.4 (181)		
Site 3	77.1 (745)	10.0 (97)	12.9 (125)			87.5 (853)	9.6 (94)	2.9 (28)		
Site 4	48.9 (244)	20.0 (100)	31.1 (155)			87.8 (430)	5.7 (28)	6.5 (32)		
Site 5	58.4 (80)	24.8 (34)	16.8 (23)			88.2 (119)	8.9 (12)	3.0 (4)		
Site 6	49.4 (129)	30.7 (80)	19.9 (52)			93.6 (247)	4.2 (11)	2.3 (6)		
**Diagnosis**										
Have cancer diagnosis	57.9 (2,124)	21.1 (774)	21.0 (772)	56.3 (6)	<.0001	91.0 (3,375)	4.5 (166)	4.6 (169)	215.9 (6)	<.0001
Have cancer & non-cancer diagnosis	61.7 (1,044)	18.5 (313)	19.8 (335)			90.0 (1,541)	6.3 (108)	3.7 (64)		
Have Non-cancer diagnosis only	73.2 (429)	13.0 (76)	13.8 (81)			75.6 (445)	20.5 (121)	3.9 (23)		
Diagnosis unspecified	59.2 (116)	25.0 (49)	15.8 (31)			86.9 (173)	9.6 (19)	3.5 (7)		

Note: df denotes degrees of freedom.

**Table 4:** **Distribution of background characteristics by fatigue and sleep disturbance performance CAPs of clients receiving palliative home care services 2006–2011, Ontario, Canada (N = 6,769)**

	Fatigue					Sleep disturbance				
	Not triggered % (N)	Trigger level 1% (N)	Trigger level 2% (N)	Chi-square (df)	p Value	Not triggered % (N)	Trigger level 1% (N)	Trigger level 2% (N)	Chi-square (df)	p Value
**Age groups**										
18-44	31 (62)	40.0 (80)	29.0 (58)	23.2 (8)	0.003	62.8 (135)	12.1 (26)	25.1 (54)	74.7 (8)	<.0001
45-64	26.3 (396)	36.3 (547)	37.4 (564)			66.2 (1,055)	12.1 (192)	21.7 (346)		
65-74	27.5 (351)	31.8 (406)	40.7 (520)			73.6 (1,015)	11.5 (159)	14.9 (206)		
75-84	25.7 (364)	34.4 (487)	39.9 (565)			76.8 (1,187)	10.0 (154)	13.3 (205)		
85 +	24.5 (136)	30.6 (170)	44.9 (249)			74.5 (462)	13.1 (81)	12.4 (77)		
**Gender**										
Male	25.3 (607)	33.9 (815)	40.9 (982)	4.7 (2)	0.09	69.5 (1,813)	13.3 (346)	17.2 (449)	20.2 (2)	<.0001
Female	27.6 (693)	34.2 (860)	38.2 (960)			74.3 (2,014)	9.7 (263)	16.0 (433)		
**Estimated prognosis**										
Death imminent	2.5 (2)	8.9 (7)	88.6 (70)	856.8 (6)	<.0001	68.8 (53)	9.1 (7)	22.1 (17)	23.1 (6)	0.0008
Less than 6 weeks	3.8 (15)	25.3 (99)	70.8 (277)			75.3 (289)	5.0 (19)	19.8 (76)		
6 weeks to 6 months	14.1 (337)	41.9 (1,000)	44.0 (1,052)			71.5 (1,635)	11.2 (257)	17.3 (394)		
Greater than 6 months	45.6 (955)	27.9 (584)	26.6 (557)			72.0 (1,483)	12.5 (258)	15.5 (320)		
**Geographic location**										
Site 1	19.5 (50)	35.2 (90)	45.3 (116)	202.3 (10)	<.0001	76.5 (199)	6.9 (18)	16.5 (43)	31.6 (10)	0.0005
Site 2	28.9 (868)	28.7 (860)	42.4 (1,273)			73.1 (2,499)	11.5 (393)	15.5 (529)		
Site 3	28.5 (262)	45.1 (415)	26.5 (244)			67.1 (633)	13.8 (130)	19.09 (180)		
Site 4	15.8 (70)	40.3 (178)	43.9 (194)			70.8 (312)	7.9 (35)	21.32 (94)		
Site 5	18.0 (20)	64.0 (71)	18.0 (20)			73.9 (68)	12.0 (11)	14.13 (13)		
Site 6	17.4 (39)	33.9 (76)	48.7 (109)			72.6 (143)	12.7 (25)	14.72 (29)		
**Diagnosis**										
Have cancer diagnosis	28.1 (823)	34.7 (1,016)	37.2 (1,090)	94.2 (6)	<.0001	73.6 (2,360)	11.3 (362)	15.1 (484)	19.8 (6)	0.003
Have cancer and non-cancer diagnosis	26.2 (375)	37.1 (530)	36.7 (525)			68.8 (1,038)	11.9 (180)	19.28 (291)		
Have non-cancer diagnosis only	17.2 (79)	24.8 (114)	58.0 (266)			71.2 (349)	9.8 (48)	18.98 (93)		
Diagnosis unspecified	23.4 (32)	21.9 (30)	54.7 (75)			71.8 (107)	14.8 (22)	13.4 (20)		

Note: df denotes degrees of freedom.

**Table 5:** **Distribution of background characteristics by delirium and mood disturbance cognition/mental health CAPs of clients receiving palliative home care services 2006–2011, Ontario, Canada (N = 6,769).**

	Delirium				Mood disturbance				
	Not triggered % (N)	Trigger level 1% (N)	Chi-square (df)	p Value	Not triggered % (N)	Trigger level 1% (N)	Trigger level 2% (N)	Chi-square (df)	p Value
**Age groups**									
18-44	91.3 (240)	8.8 (23)	12.9 (8)	0.02	63.4 (156)	14.2 (35)	22.4 (55)	45.2 (8)	<.0001
45-64	86.8 (1,601)	13.2 (244)			61.4 (1,105)	17.5 (315)	21.2 (381)		
65-74	86.1 (1,348)	13.9 (217)			63.8 (953)	18.0 (269)	18.2 (272)		
75-84	87.1 (1,540)	12.9 (228)			68.1 (1,161)	16.2 (276)	15.7 (267)		
85 +	83.1 (580)	16.9 (118)			71.7 (492)	16.0 (110)	12.2 (84)		
**Gender**									
Male	86.0 (2,567)	14.0 (417)	1.3 (2)	0.25	66.1 (1,904)	16.6 (479)	17.2 (496)	2.2 (2)	0.33
Female	87.0 (2,706)	13.0 (403)			64.3 (1,933)	17.4 (522)	18.3 (551)		
**Estimated prognosis**									
Death Imminent	30.5 (25)	69.5 (57)	510.5 (6)	<.0001	65.1 (54)	19.3 (16)	15.7 (13)	73.3 (6)	<.0001
Less than 6 weeks	62.1 (267)	37.9 (163)			51.3 (205)	18.5 (74)	30.3 (121)		
6 weeks to 6 months	85.5 (2,171)	14.5 (368)			62.1 (1,492)	18.3 (440)	19.6 (470)		
Greater than 6 months	93.3 (2,036)	6.7 (146)			69.6 (1,490)	15.3 (328)	15.1 (324)		
**Geographic location**									
Site 1	79.6 (214)	20.5 (55)	85.1 (10)	<.0001	62.3 (167)	15.3 (41)	22.4 (60)	34.9 (10)	0.0001
Site 2	89.1 (3,560)	10.9 (437)			66.9 (2,666)	17.2 (684)	16.0 (638)		
Site 3	81.6 (791)	18.5 (179)			62.0 (591)	15.3 (146)	22.8 (217)		
Site 4	80.9 (402)	19.1 (95)			62.9 (251)	18.3 (73)	18.8 (75)		
Site 5	93.6 (131)	6.4 (9)			61.1 (58)	17.9 (17)	21.1 (20)		
Site 6	79.3 (211)	20.7 (55)			59.0 (134)	19.4 (44)	21.6 (49)		
**Diagnosis**									
Have cancer diagnosis	88.0 (3,221)	12.0 (438)	42.5 (6)	<.0001	66.8 (2,336)	16.2 (566)	17.0 (595)	31.4 (6)	<.0001
Have cancer and non-cancer diagnosis	86.0 (1,482)	14.0 (241)			60.5 (1,017)	18.4 (309)	21.1 (355)		
Have non-cancer diagnosis only	78.1 (449)	21.9 (126)			67.5 (382)	18.7 (106)	13.8 (78)		
Diagnosis unspecified	86.3 (157)	13.7 (25)			70.6 (132)	12.8 (24)	16.6 (31)		

Note: df denotes degrees of freedom.

The prevalence of persons who triggered the Dyspnea CAP generally increased with age, increased as the estimated prognosis was shorter, and was higher in females. In addition, persons with a non-cancer diagnosis were significantly more likely to trigger the Dyspnea CAP compared to persons with a cancer diagnosis (62.3% vs. 42.2% p < 0.0001). CCAC Site 5 reported substantially lower rates of persons who triggered the Dyspnea CAP than other CCAC sites (23.6% for CCAC Site 1 vs. range from 37.5% in CCAC Site 1 to 46.87% in CCAC Site 2).

The prevalence of persons who triggered the Nutrition CAP also increased as the estimated prognosis was shorter and was more common in females. An exception to the overall increase in triggering of the Nutrition CAP with increased age overall was, a curvilinear relationship observed among persons who triggered level one (low BMI) of the Nutrition CAP where the youngest (aged 18–44) and the oldest old (85+) age groups were more likely to trigger. For the Nutrition CAP, although overall triggering prevalence’s were comparable across sites, persons from the CCAC Site 5 reported the highest triggering rates at level one (17.5%) but the lowest prevalence at level two (9.7%). In contrast, CCAC Site 1 exhibited the lowest triggering rates for the Nutrition CAP at level one (4.7%) and the highest prevalence of Nutrition CAP triggering rates at level two (21.1%).

The prevalence of persons who triggered the Pain CAP was highest for persons aged 18–44 and generally decreased with age. Prevalence of triggering the Pain CAP increased as the estimated prognosis was shorter. No differences were observed in prevalence of Pain CAP triggering by gender. Persons with a cancer diagnosis were significantly more likely to trigger the Pain CAP compared to persons with a non-cancer diagnosis (42.1% vs. 26.8% p < 0.0001). Variation in Pain CAP triggering ranged substantially from a low of 22.9% in CCAC Site 3 to more than double that in CCAC Site 4 (51.1%).

Persons over the age of 65 exhibited were more likely to trigger the Pressure Ulcers CAP and more specifically, most likely to trigger at level 1 (Difficult to improve). No differences were observed in prevalence of Pressure Ulcers CAP triggering by gender. Persons with a non-cancer diagnosis were significantly more likely to trigger the Pressure Ulcers CAP compared to persons with a cancer diagnosis (24.4% vs. 9.0% p < 0.0001). Geographic variation in Pressure Ulcer CAP triggering ranged from 6.4% in CCAC Site 6 to 12.5% in CCAC Site 3.

The prevalence of persons who triggered the Fatigue CAP increased with age (Table 4). It also increased as the estimated prognosis was shorter from 54.4% among those with a prognosis greater than 6 months to a prevalence of 97.5% for those whose death was imminent. The number of persons who triggered the Fatigue CAP at level two (high risk) nearly tripled from 26.6% for those with an estimated prognosis of greater than six months to 88.6% when death is imminent. No significant differences were evident by gender. Persons with a non-cancer diagnosis were significantly more likely to trigger the Fatigue CAP compared to persons with a cancer diagnosis (82.8%% vs. 71.9% p < 0.0001). Prevalence of Fatigue CAP triggering ranged greatly by site where CCAC Site 2 reported the lowest rates for Fatigue CAP triggering (71.9%) compared to a high of 83.8% reported by CCAC Site 4.

The prevalence of persons who triggered the Sleep Disturbance CAP increased with age (Table 4). While similar rates were observed by age for triggering the Sleep Disturbance CAP at level one (moderate potential to improve), 25.1% of younger persons (aged 18–44) reported triggering the Sleep Disturbance CAP at level two (high potential to improve) double that reported by the oldest old (12.4%). Prevalence of triggering the Sleep Disturbance CAP was higher for males, persons with a shorter estimated prognosis, and persons with both a cancer and non-cancer diagnosis. Geographic variation in Sleep Disturbance CAP triggering rates ranged by CCAC site from 31.2% in CCAC Site 3 to low of 23.5% in CCAC Site 1.

A general increase in Delirium CAP triggering is shown by age. Persons aged 85 or greater exhibited the highest triggering rate, nearly double the rate of those aged 18–44. The Delirium CAP is most commonly triggered by persons with a shorter prognosis, with over two-thirds triggering the Delirium CAP when death is imminent. Those with a non-cancer diagnosis were more likely to trigger the Delirium CAP than those with cancer. Site variations in Delirium CAP triggering ranged greatly from 6.4% in the CCAC Site 5 (n = 9) to over 20% in CCAC Site 1 (n = 55).

Prevalence of Mood Disturbance CAP triggering frequency decreased with age with the exception for youngest age group. However, among those who triggered the Mood Disturbance CAP, younger persons were more likely to trigger at a level two. The Mood Disturbance CAP was most commonly triggered by persons with an estimated prognosis of less than 6 weeks. Variation in Mood Disturbance CAP triggering rates was less than 8% between CCACs (59.0% in CCAC Site 6 to 66.9% in CCAC Site 2). Persons with a cancer and non-cancer diagnoses triggered the Mood Disturbance CAP more frequently than those with only a cancer or only a non-cancer diagnosis.

**Figure 3:**
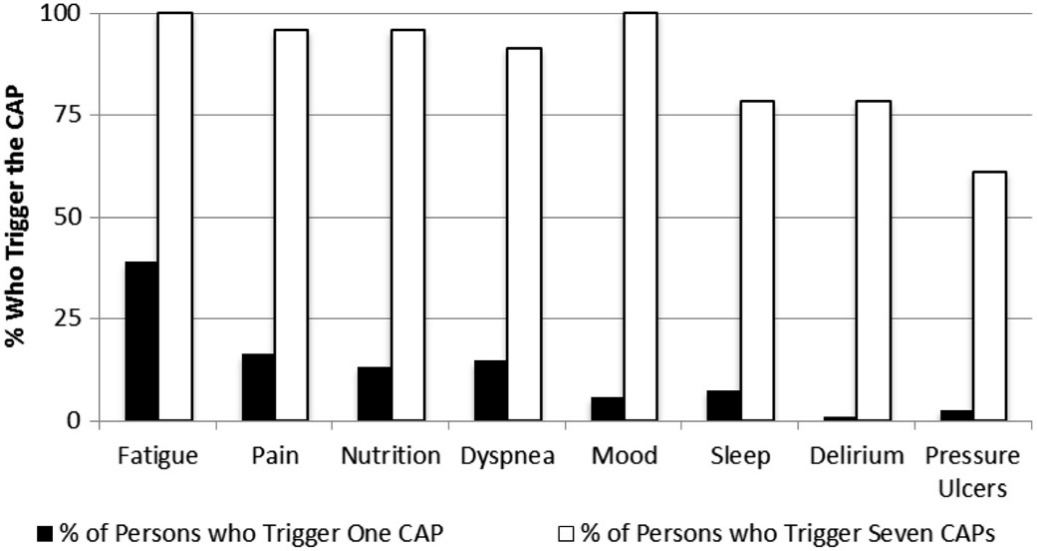
**CAP Triggering rates by number of CAPs triggered of palliative home care clients 2006–2011, Ontario, Canada (N = 6,769).**

Through examination of the count of triggered CAPs a hierarchical structure in triggering emerged (Figure 3). Fatigue was the most commonly triggered CAP, triggered by 38.9% of persons who triggered only one CAP to over 90% of persons who triggered three to five CAPs and 100% of persons to triggered six or more CAPs. Captured in the percentage of persons who trigger only one CAP, the Fatigue CAP, Pain CAP, Nutrition CAP, and Dyspnea CAP emerged as early-triggered CAPs. In contrast, the Delirium and Pressure Ulcer CAPs emerged as late-triggered CAPs. Consequently, persons who triggered only one CAP, were most likely to trigger the Fatigue, Pain, Nutrition, or Dyspnea CAPs and were least likely to trigger the Delirium CAP and Pressure Ulcer CAP. Among those who triggered seven CAPs, all persons triggered the Fatigue and Mood Disturbance CAPs and over 90% triggered the Dyspnea, Nutrition, and Pain CAPs. In contrast, the Sleep Disturbance, Delirium, and Pressure Ulcers CAPs were triggered less frequently. When all but one CAP were triggered, the Sleep Disturbance, Delirium, and Pressure Ulcers CAPs remained least likely to be triggered.

## Discussion

Analyses of interRAI PC CAP triggering rates in Ontario, Canada illustrate the information to be gained from the interRAI PC, a comprehensive standardized assessment instrument. Covering especially pertinent clinical issues, performance, and mental health/cognition domains, the eight interRAI PC CAPs emphasize need for care planning in key areas of palliative care. The majority of persons triggered two or more CAPs reflecting high levels of clinical need within the palliative home care sample. Variation in CAP triggering was evident based on the age, estimated prognosis, geographic location and diagnosis of the person. Older persons and those with a shorter estimated prognosis were most likely to trigger multiple CAPs.

As emphasized by the WHO, comprehensive assessment is an integral component of quality palliative care. White, McMullan, and Doyle found that two thirds of symptoms experienced by persons receiving palliative care services were not immediately self-reported [30]. Instead, the majority of symptoms were detected through systematic questioning during assessment. As fatigue and dyspnea emerged as early-triggering CAPs, it is suggested that systematic questioning of these symptoms be prioritized during all clinical assessments. Treatment of symptoms at end of life can improve the QOL of the person and their informal support network [31]. The increased health complexity of persons requiring palliative care necessitates individualized care planning. Decision making strategies informed by evidence from the interRAI PC CAPs assist clinicians in developing a person-centered care plan, identifying areas of need, and prioritizing treatment options in consultation with the person.

Evidence of a hierarchical structure in CAP triggering may be useful to predict health complexity and change over time. For persons with multiple health concerns, the high frequency of Fatigue CAP, Dyspnea CAP, Pain CAP, Nutrition CAP, and Mood Disturbance CAP triggering warrants increased awareness on the part of the clinician and other caregivers. The Fatigue CAP and Dyspnea CAP emerge as pervasive issues among the overall palliative home care sample. When persons seem relatively stable with few major health concerns, the hierarchical nature of CAP triggering suggests clinicians should continue to investigate fatigue and dyspnea. In contrast, the Delirium CAP and Pressure Ulcers CAP trigger at higher frequency for persons nearing end of life. The Delirium CAP and Pressure Ulcers CAP, late-triggering CAPs, rarely trigger in isolation and may highlight increased client need. The late-triggering of the Delirium CAP and Pressure Ulcers CAP suggest they are indicative of later stages of need in palliative care. Further investigation into the role of CAP triggering and symptom clusters is needed. The ability of the CAPs to identify symptoms also requires further assessment.

Consistent with previous research, the majority of palliative care clients in this study (86.8%) reported a diagnosis of cancer. Seow, King and Vaitonis note that although persons with cancer receive 80-85% of palliative care services in Ontario, they account for only one third of persons who die during the time of the study [32]. Research used to inform current palliative care practice focuses almost exclusively on the needs of persons with a cancer diagnosis [33]. Benefits of palliative care for persons with a cancer diagnosis and their informal support network during the rapid decline phase preceding death are well recognized. Palliative care has been best known to benefit persons with cancer during the last few months of life [34, 35].

However, findings from this study stress the need to broaden understanding of how persons without cancer receiving palliative care may differ. Disease diagnosis, more specifically the presence or absence of a cancer diagnosis, was a strong predictor of health characteristics and CAP triggering among persons at the end of life. Persons reporting only a cancer diagnosis were more likely to trigger the Pain CAP. This may be expected, as there is increased awareness of the benefits of palliative care to address pain for persons with cancer and addressing uncontrolled pain is often a reason for referral. Therefore, persons with cancer who are experiencing challenges with pain may be more likely to be referred for palliative care services.

In contrast, those reporting only non-cancer diagnoses such as heart failure, stroke, COPD, or dementia, were significantly more likely to trigger the Dyspnea CAP, Nutrition CAP, Pressure Ulcers CAP, Fatigue CAP, and Delirium CAP. This suggests that persons with non-cancer diagnoses, who access palliative care services in Ontario, are more likely to exhibit increased health complexity and require person-specific tailoring of interventions to address multiple symptoms. Persons who reported both a cancer and non-cancer diagnosis were most likely to trigger the Mood Disturbance and Sleep Disturbance CAPs. For the Mood Disturbance CAP, the prevalence of triggering with a single symptom was equally as high for those with non-cancer and cancer diagnoses and for persons with non-cancer diagnosis only. However, those with both a cancer and non-cancer diagnosis were much more likely to trigger at a level 2. The level of psychosocial distress may be comparable between persons with non-cancer and cancer diagnoses [36]; however the present results suggest this burden may be amplified when other conditions are present. This is also reflected in the CAP hierarchical triggering structure. The Mood Disturbance CAP was not commonly triggered alone, but when almost all CAPs were triggered, all persons triggered the Mood Disturbance CAP. This suggests that triggering the Mood Disturbance CAP may be related to increased symptom burden. It may also be possible that symptom characteristics such as length of time since onset, intensity, and frequency in relation to disease diagnosis may also affect the degree the symptoms impact on the person’s health and QOL.

Palliative care should respond to the needs of persons of all ages. Findings from this study suggest that older persons are not only more complex and likely to exhibit the greatest needs but that with the exception of the Pain CAP and Mood Disturbance CAP, they trigger CAPs more frequently. Age-related barriers to palliative care referral, resource allocation, and service utilization in Canada have been reported elsewhere [37–40] and may be compounded by other challenges such as disease diagnosis, and geographic location of care [41]. Therefore, greater investigation into how age affects patterns in CAP triggering is warranted.

While the majority of CAPs were more likely to be triggered among older cohorts, two exceptions were the Mood Disturbance CAP and Pain CAP which younger persons were most likely to trigger. A reason for these discrepancies may be that mood disturbances and pain are not less prevalent in younger age groups but rather that challenges exist for clinicians to recognize these symptoms [42, 43]. Difficulties with mood and in particular symptoms of depression may also be under-recognized in the older adult population due to their atypical presentation [21, 44]. Mood disturbances and depression among older adults may be expressed as physical rather than psychological symptoms such as fatigue, weight loss, or gastro-intestinal problems in contrast to direct communications of feelings of sadness or expressions of depressed mood. This presents unique age-associated challenges for clinicians to recognize the signs of depression and mood disturbance in an older adult population.

The Pain CAP is commonly triggered for those aged 18–44. There are many possible explanations for this. First, younger persons experiencing severe pain or challenges in pain management may feel more confident to voice their concerns over pain management and therefore be referred more often than older adults who may be more hesitant to discuss pain symptoms [45]. The ageist myth, that older adults are used to pain and do not need treatment, may result in pain not being noted or addressed. Older adults may be hesitant to express feelings of pain due to the belief that it is a natural part of the aging process [45]. Rao and Cohen note that lack of recognition of pain symptoms and severity, as well as a lack of understanding of the benefits of pain treatment and management is frequently seen in older persons [46]. Cognitive impairment may affect the ability to communicate pain, challenge the clinician’s ability to recognize signs that pain is present, and result in the under-reporting of pain [47]. However, it may also reflect a failure of clinicians to recognize pain among the older population. The prevalence of persons exhibiting cognitive impairment increases with age and thereby may also result in an elevated risk for under-recognition and under-treatment of pain for older adults.

Conversely, limitations of the present study should also be recognized. First, the sample is based on volunteer organizations representing some, but not all, regions of Ontario, meaning the results may not be generalizable to the full provincial population or to other jurisdictions. Second, given that the data were drawn from pilot studies prior to the mandated implementation in Ontario there were some issues with missing data for certain interRAI PC items that would generally not occur when a formal reporting system (e.g., Canadian Institute for Health Information’s Home Care Reporting System) is in place. This meant that some items that may have been helpful for creating CAP triggers were excluded due to missing data. The section most affected by this issue was the item set dealing with spirituality. Finally, date of death was available only for a subset of pilot study participants so it was not possible to examine associations of CAPs with survival time. This may be addressed in future research because the instrument has now been implemented province wide as the standard assessment for this sector and the data are linkable to administrative records that would include date of death.

It is commonly accepted that as persons near the end of life, the number of health issues and challenges increases. CAP triggering rates differ greatly by age and estimated prognosis. The CAPs do not provide a set treatment plan, but help guide the clinician to consider relevant issues, assist in the prioritization of treatment feasibility, and inform best practice guidelines. In consultation with the person and, when appropriate, members of their informal support network, decisions on whether or not issues raised by the CAPs should be addressed must be made on a case-by-case basis. Wishes expressed by the person should be reflected when assessing treatment burden-benefit and determining whether or not to treat. It is important to remember that even in the final stages of life, persons may respond to and benefit from treatments that decrease symptom burden thereby improving QOL at the end of life.

## Conclusion

Data gathered from the interRAI PC may improve the understanding of the complex needs of palliative home care clients in Ontario and other locations. Patterns in CAP triggering suggest increased attention should be given to address the increasingly complex needs of vulnerable populations. Future research should investigate variation by geographic location and reasons for age-associated disparities in CAP triggering. Integration of evidence gathered from the interRAI PC CAPs into the care planning process may allow for higher quality of care through better tailoring of resources at address person-specific need.
